# The German pediatric integrated care survey (PICS-D): Translation, adaptation, and psychometric testing

**DOI:** 10.3389/fped.2022.1057256

**Published:** 2022-12-23

**Authors:** Jana Willems, Isabella Bablok, Matthias Sehlbrede, Erik Farin-Glattacker, Thorsten Langer

**Affiliations:** ^1^ Section of Health Care Research and Rehabilitation Research, Faculty of Medicine and Medical Center, University of Freiburg, Freiburg, Germany; ^2^ Department of Neuropediatrics and Muscle Disorders, Center for Pediatrics, Faculty of Medicine, University of Freiburg, Freiburg, Germany

**Keywords:** chronic condition, reported outcome measures, integrated care, psychometric properties, chronic disease

## Abstract

**Background:**

Integrated care models aim to strengthen the collaboration between healthcare sectors to ensure a higher quality of care for children with chronic conditions. Assessing pediatric healthcare integration through families' experiences, therefore, is essential. Our study aimed to psychometrically test the PICS-D, the first German-language integrated care questionnaire, which is based on the Pediatric Integrated Care Survey (PICS) developed in the USA.

**Methods:**

We examined construct validity using exploratory and confirmatory factor analyses (structural validity). Cronbach's *α* and McDonald's *ω*_t_ coefficients explored reliability. Cognitive interviews assessed wording comprehension and item appropriateness.

**Results:**

PICS-D was completed by 204 caregivers of children with chronic conditions (women = 84%; mean age = 41.4 years). Factor analyses identified 3 factors: “Team quality & communication”, “Family impact”, and “Access to care”. The 3-factorial structure showed a satisfying fit to confirmatory classical-test-theory-based models. Due to the insufficient reliability of the third factor, we recommend using only factors 1 and 2 for scoring the PICS-D.

**Conclusion:**

The PICS-D is a 13-item questionnaire to assess family-reported experiences in pediatric care integration, which has good psychometric properties. It may be useful in guiding quality improvement efforts or measuring the impact of a care plan or care model.

**Trial registration**: German Clinical Trials Register (DRKS): DRKS00018778; Trial registration date 05. December 2019—Retrospectively registered; https://apps.who.int/trialsearch/Trial2.aspx?TrialID=DRKS00018778.

## Introduction

One of the most persistent problems in many healthcare systems is the fragmentation between sectors and disciplines, which particularly affects patients with chronic and complex conditions who rely on services from a variety of disciplines (1–6). Integrated care (IC) models aim to strengthen the collaboration between sectors to ensure a higher quality of care (2, 7). The definition of IC varies widely, focusing primarily on adult care (8, 9). Tailoring these definitions to the pediatric setting is essential, as children have different healthcare and social needs (4, 10). By integrating the child's health perspective, Antonelli et al. extend existing definitions and offer the framework upon which this study is based: “*Integrated Care is the seamless provision of healthcare services, from the perspective of the patient and family, across entire care continuum. It results from coordinating the efforts of all providers, irrespective of institutional, departmental, or community-based organizational boundaries*.” (11–13). This definition implies that patient and family experiences are relevant when assessing IC in pediatric settings (6, 14). To date, evidence on the effectiveness of existing IC models (especially in adult care) has been predominantly specific for certain conditions (4, 8, 15–17). Other pediatric questionnaires, such as the EMPATHIC-30, cover only single facets of care integration (e.g., family-centered care experiences) and are limited to individual pediatric disciplines (18, 19). Generic evaluations often fail to adequately define goals and outcomes, and provide too little information on the availability of established measurement instruments (8, 11–13, 20). The Pediatric Integrated Care Survey (PICS) is a questionnaire for the caregivers of chronically ill children which measures the family-reported experience of pediatric care integration in the US (21). In German-speaking countries, instruments to measure IC currently exist only in the field of adult care (22–26)—a fact highlighting the need for a validated questionnaire to assess how well integrated pediatric healthcare is for families.

## Materials and methods

### Overview of the pediatric integrated care survey (PICS)

Antonelli et al. at Boston Children's Hospital developed the PICS in 2013. They formed and guided focus groups and interviews with parents of chronically ill children receiving care from multiple medical and non-medical providers (11, 21). It consists of 19 validated (mainly Likert-scaled) core items that form five scales (e.g., Access to care, Family impact, and Team functioning/performance/quality/connectivity). An example item in the “Family impact” scale is as follows: “In the past 12 months, how often have your child's care team members talked with you about how healthcare decisions for your child will affect your whole family?”. The six-point response scale ranges from “never” to “always”. The authors propose a 12-month reference period to evaluate IC. There are supplementary items that have not been validated due to sample size limitations in the Ziniel et al. study, as well as accompanying demographic and healthcare status questions (21). The supplementary items are grouped into so-called “modules” and “supplementary question topics”. Antonelli et al. recommend selecting items in the set of supplementary questions in addition to the validated core items, depending on the focus of use (11).

### Research aims

This study is part of the exploratory, prospective, controlled, two-armed SMA-C + -study, developed as an IT-supported Case Management to improve the care of patients with spinal muscular atrophy I and II (27). In this comprehensive project, we use the German version of the PICS to evaluate the Case Management as an IC intervention. Furthermore, we evaluated the questionnaire's psychometric properties by testing construct validity (as factorial/structural validity) and internal consistency. We, therefore, included the set of core items that were already validated in the English version.

### Pre-data collection methods

The original PICS items' translation followed the six steps in the “Functional Assessment of Chronic Illness Therapy Translation Procedures and Guidelines” (FACIT): First, two native German speakers translated the instrument independently into German. A third native German speaker reviewed the translations and produced a preliminary version. A native English speaker then translated the preliminary version back into English. In subsequent steps, methodological and bilingual experts evaluated and discussed the different versions. In the final revision, the translated instrument is checked orthographically by a bilingual expert (28).

We conducted cognitive semi-structured interviews to assess wording comprehension and the item appropriateness of the preliminary PICS-D from the caregiver's perspective (29). For this purpose, we recruited *N* = 10 caregivers of chronically ill children in the Clinic of Neuropediatrics and Muscle Disorders of the University Medical Center Freiburg. After seven interviews, we revised problematic items based on their feedback. We then conducted another three interviews.

### Pilot testing

We used the PICS-D for a cross-sectional study of family-reported experiences with IC involving caregivers of children with a chronic disease. Taking the original authors' approach, we identified patients with chronic conditions based on their care needs. Patients are categorized as having a chronic condition if at least one of the following criteria is met: regular use of medications, regular use of therapies, use of auxiliary aids, regular counseling, or psychotherapy. We recruited *N* = 204 caregivers Germany-wide *via* personal contact from the University Medical Center, Freiburg; the Clinic for Pediatrics I, University Hospital, Essen; and several social pediatric centers (SPC). SPCs are interdisciplinary outpatient clinics that provide multidisciplinary care for children with complex healthcare needs. The participating SPCs are located in Bochum, Bremen, Celle, Freiburg, Lübeck, Rotenburg (Wuemme) and, Stuttgart. Caregivers were approached individually and were given the questionnaire and an information leaflet. In addition, the patient organization “Children's Network Germany” posted an invitation to participate on its website. The “Children's Network” represents pediatric patient organizations in Germany. All participants received a 20 € voucher per completed questionnaire. Exclusion criteria for participating in the questionnaire were not having a chronically ill child, limited German proficiency, or a patient's or caregiver's refusal to participate. Data collection took place between March 2019 and May 2020.

### Statistical analyses

The statistical methods applied in psychometrically testing the PICS rely on the procedure described in the original publication and are supplemented by analyzing additional properties (21). Antonelli et al. recommend a top/bottom box scoring (11). The top/bottom boxes are the highest and lowest ratings (most positive/negative rating) on a response scale. Therefore, the item is “transformed” into a binary variable, and the respondent receives a box score of 1 indicating that he/she checked the desirable answer (most positive/negative answer). In contrast, we did not dichotomize the items and considered the response scale as an interval-scaled variable. The original authors indicate that the recommended scoring method is primarily for communicating quality improvement to healthcare institutions/professions, which is not a primary endpoint of our study. We described sample characteristics for children and respondents (caregivers) and assessed distribution properties, structural validity, and internal consistency (Cronbach's *α* and McDonald's *ω_t_*). We performed descriptive analyses of the sample using IBM SPSS® and applied factor analyses in R (30).

Due to the content adjustment of the PICS-D, we refrained from confirmatory testing of the factor structure of the original PICS and conducted two exploratory factor analyses (EFA; extraction method: principal axis factoring after direct oblimin rotation) to identify concepts underlying the IC experience in German pediatric settings. Kaiser-Mayer-Olkin (KMO) coefficient and Bartlett sphericity test were used to analyze the suitability of the data for EFA. We calculated different criteria to determine the number of retained factors [Kaiser-Guttman criterion, parallel analysis and Velicer's MAP (31)]. We excluded three items on goal setting and access to medical records (original items 13, 30, and 31) from the beginning because the response scale deviated from a Likert scale (e.g., yes/no response format). Therefore, we included 16 Likert-scaled experience items (instead of the 19 original items) in the first EFA and 13 Likert-scaled items in the second EFA (another three items were excluded during the analysis process). We recoded response options such as “I don't know.” or “I have no concerns.” as missing values. Because the first EFA was mainly intended to provide exploratory support for selecting items, only the second EFA's results are reported in detail below.

To further analyze the PICS-D structure, we conducted two confirmatory factor analyses (CFA) using confirmatory classical-test-theory-based models. In each case, the CFAs followed an EFA that had been already been performed. We used the first CFA to obtain statistical criteria to exclude further items. The second CFA tested the performance of the final factor structure. Global measures of fit indicate the discrepancy between the data and hypothetical model (CFI, TLI, RMSEA) (32, 33). Bad fit of models may indicate violation of unidimensionality (i.e., item not related to the underlying concept) or insufficient item reliability. To determine the best dimensional structure, we conducted the CFAs on each factor as well as the whole model. Furthermore, we explored each factor's internal consistency *via* Cronbach's *α* and McDonald's *ω_t_* coefficient.

## Results

### Translation and adaptation

This section describes modifications to the original core items for assessing pediatric care integration in Germany. Table 1 shows the original 19 core items in both German and English translations (note: the latter does not match the wording of the original PICS items due to adaptations to the German health care system). The assignment of the original items, as well as their respective numbering, is found in the PICS “User Manual” (11). Most of the item changes resulted from the need to linguistically simplifying the wording, or adding information (concrete examples) for clarification. To ensure that the originally intended meaning of an item's wording remained intact, we consulted with the original authors at regular intervals. Moreover, cognitive interviews showed that the wording “care team” created confusion between the concepts of a “fixed team”, that works cohesively as a unit and a team that results from different responsibilities within the child's care. Caregivers reported that the care of their children had no “fixed team”. That is why we added an instruction defining the care team as all individuals involved in caring for the chronically ill child in the past 12 months (called the “care network”). After finalizing the questionnaire, we discussed the items with the original authors *via* videoconference. The translated PICS-version contained 19 core items on experiences (Table 1), a demographic and descriptive section including 32 items, and one open-ended item.

**Table 1:** Translation of the 19 core items of the PICS-D.

No. of item in original PICS	Item wording of the PICS-D	Translated item wording of the PICS-D
13**	Hatten alle Behandelnden Zugang zu den für sie wichtigen medizinischen Unterlagen (z.B. Arztbriefe)? Wie ist Ihre Einschätzung?	Did all medical providers have access to the medical records that were important to them (e.g., doctors’ letters)? What is your opinion?
17*	Wie oft hatten Sie Schwierigkeiten bzgl. medizinischer oder sozialer Dienste (z.B. Therapien, Integrationshilfe etc.), weil es Wartelisten oder andere Probleme bei der Terminvergabe gab?	How often did you have difficulties getting medical or social services (e.g., therapies, integration assistance, etc.), because there were waiting lists or other problems getting appointments?
18*	Wie oft hatten Sie Schwierigkeiten bzgl. medizinischer oder sozialer Dienste (z.B. Therapien, Integrationshilfe etc.), weil Sie nicht wussten, wer der richtige Ansprechpartner ist?	How often did you have difficulties getting medical or social services (e.g., therapies, integration assistance, etc.), because you did not know whom to contact?
22*	Wie oft haben die Mitglieder des Versorgungsnetzes Ihnen Dinge so erklärt, dass Sie sie verstehen konnten?	How often have the care network members explained things to you in a way that you could understand?
23*	Wie oft hatten Sie das Gefühl, dass Behandlungsempfehlungen zwischen den Mitgliedern des Versorgungsnetzes weitergegeben wurden?	How often did you feel that treatment recommendations were passed between the members of the care network?
24*	Manchmal haben Eltern Bedenken zur Gesundheit und Versorgung Ihres Kindes. Wie oft ist es Ihnen leicht gefallen, den Mitgliedern des Versorgungsnetzes Ihre Bedenken mitzuteilen?	Sometimes parents have concerns about their child's health and care. How often did you feel comfortable to share your concerns with the care network members?
25*	Wie oft hatten Sie das Gefühl, dass Sie von den Mitgliedern des Versorgungsnetzes gehört wurden, wenn Sie etwas über die Gesundheit Ihres Kindes zu sagen hatten?	How often did you feel that the members of the care network listened to what you had to say about your child's health?
26*	Wie oft wurde mit Ihnen besprochen, wer für die verschiedenen Bereiche der Versorgung Ihres Kindes verantwortlich ist?	How often has someone explained to you who was responsible for different parts of your child's care?
27*	Wie oft hatten Sie das Gefühl, dass die Mitglieder des Versorgungsnetzes über alle Tests und Untersuchungen Ihres Kindes informiert waren, um unnötige Untersuchungen zu vermeiden?	How often did you feel that the care network members were aware of all tests and evaluations of your child in order to avoid unnecessary testing?
28*	Wie oft hatten Sie das Gefühl, dass die Mitglieder des Versorgungsnetzes ihre Aufgaben in der Versorgung Ihres Kindes erfüllten?	How often did you feel that the care network members were fulfilling their roles in your child's care?
29*	Wie oft hatten Sie das Gefühl, dass die Mitglieder des Versorgungsnetzes bei der Betreuung Ihres Kindes seine Gesamtsituation berücksichtigt haben, d.h. alle Bedürfnisse Ihres Kindes?	How often have you felt that care network members thought about the “big picture” when caring for your child, meaning dealing with all of your child's needs?
30**	Haben die Mitglieder des Versorgungsnetzes kurzfristige Behandlungsziele für Ihr Kind gesetzt, d.h. Ziele bis zu 6 Monaten in der Zukunft?	Have the care network members created short-term care goals, meaning goals up to 6 months in the future?
31**	Haben die Mitglieder des Versorgungsnetzes langfristige Behandlungsziele für Ihr Kind gesetzt, d.h. Ziele, die 6 Monate oder länger in der Zukunft liegen?	Have the care network members created long-term care goals, meaning goals that are 6 months or more in the future?
32*	Wie oft haben die Mitglieder des Versorgungsnetzes Sie als vollwertige/n Partner/in behandelt?	How often have the care network members treated you as a full partner?
33*	Wie oft haben die Mitglieder des Versorgungsnetzes mit Ihnen darüber gesprochen, wie sich Entscheidungen über die Versorgung Ihres Kindes auf Ihre ganze Familie auswirken werden?	How often have the care network members talked with you about how care decisions for your child will affect your whole family?
34*	Wie oft haben die Mitglieder des Versorgungsnetzes mit Ihnen über Belastungen gesprochen, die sich für Sie durch die Erkrankung Ihres Kindes ergeben können?	How often have members of the care network talked with you about burdens you may face as a result of your child's illness?
35*	Wie oft haben die Mitglieder des Versorgungsnetzes Situationen angesprochen, die es Ihnen schwer machen können, sich um die Gesundheit Ihres Kindes zu kümmern (z.B. Arbeit, eigene Krankheit etc.)?	How often have the care network members addressed situations that may make it difficult for you to care for your child's health (e.g., work, own illness, etc.)?
36*	Wie oft haben die Mitglieder des Versorgungsnetzes angeboten, mit Ihnen auf andere Weise als durch einen persönlichen Besuch zu kommunizieren (z.B. Telefon, E-Mail oder Skype), wenn keine körperliche Untersuchung erforderlich war?	How often have the care network members offered to communicate with you in ways other than an in-person visit, such as phone, email, skype, if no physical examination was necessary?
37*	Wie oft haben Ihnen die Mitglieder des Versorgungsnetzes die Möglichkeit angeboten, mit anderen betroffenen Familien in Kontakt zu treten?	How often have the members of the care network offered you the opportunity to connect with other affected families?

* Items scored from 1 to 6: 1 = “never”; 2 = “rarely”; 3 = “sometimes”; 4 = “usually”; 5 = “almost always”; 6 = “always”.

** Additional items: not included in scoring, with two answer modalities: “yes” “no”.

### Sample characteristics

204 caregivers of children with chronic conditions completed the PICS-D, 84.3% of participants were female, 14.7% male. Two-thirds of respondents were between 30 and 50 years old and married. Slightly more than half of the participants were employed either part-time, not employed or not capable of gainful employment. The vast majority of the respondents' chronically ill children were between 1 and 12 years old. In 47% of participants, 2–5 health care providers were involved in the child's care. For around one-third of respondents, their care network even consisted of 6–10 health care providers. Further characteristics are detailed in Table 2.

**Table 2:** Child and caregiver descriptive characteristics.

Variable	%
**Respondent gender (*n* = 202)**	
Female	84.3
Male	14.7
**Respondent age at questionnaire completion (*n* = 201)**	
<30 years.	7.5
30–40 years.	40.8
40–50 years.	35.3
>50 years.	15.4
**Respondent relationship to child (*n* = 203)**	
Mother	80.4
Father	13.7
Other	5.5
**Respondent education (*n* = 203)**	
Primary school. secondary school and secondary modern	10.3
High school	6.4
Completed training	38.4
University degree (Bachelor. Master. Doctorate)	22.7
Other	22.3
**Respondent family status (*n* = 202)**	
Single	5.4
Married	77.7
Living in a steady partnership	5.4
Divorced. separated	10.4
Widowed	1.0
**Respondent employment status (*n* = 201)**	
Employee full-time	18.4
Employee part-time	41.8
Civil servant	6.0
Self-employed	8.5
Not gainfully employed or capable of gainful employment	15.4
Other	10.0
**Child gender (*n* = 204)**	
Female	49.5
Male	50.5
**Child age at questionnaire completion (*n* = 202)**	
<1 year.	2.5
1–3 years.	20.3
3–12 years.	44.1
12–18 years.	24.3
>18 years.	8.9
**Number of healthcare providers involved in the care of the child (*n* = 202)**	
2–5	47.0
6–10	38.1
11–15	10.4
>15	4.5
**Health insurance (*n* = 199)**	
Statutory	75.4
Statutory with additional insurance	12.1
Private	12.1
I do not know.	0.5
**Primary language spoken at home (*n* = 203)**	
German	85.2
Other	14.8

### Structural validity

The KMO coefficient (KMO = .89) and Bartlett sphericity test (*χ*^2^ = 1427.29, *p* < .001) indicated that the data we collected are suitable for the EFA. Parallel analysis and Kaiser-Guttman criterion suggest a three-factor solution, MAP two factors. We compared both EFA models and selected the three-factor solution because of a substantial better fit (RMSA < .08, SRMR < .05) in comparison to the two-factor solution. The three factors solution explained 53% of variance with low factor loadings on item 24 (share concerns with care network) and item 36 (communicate in ways other than an in-person visit). Factor loadings for the two items were item 24: 0.23, 0.19, and 0.10 in factors 1, 2, and 3, respectively and item 36: 0.33, 0.26, and 0.03 in factor 1, 2, and 3, respectively. Cognitive interviews revealed that both items were poorly understood due to their length and complicated syntax and we therefore excluded these items from our analysis. After exclusion, we conducted a first CFA for factor 1 and 2 that revealed high residual correlation within factor 1 for the item 27 with the items 23 and 25. Based on statistical and clinical considerations, we thus eliminated that item and then repeated the EFA and CFA analysis with 13 items.

The three factors of the second EFA for the PICS-D items explained 58% of the variance. The items' standardized loadings were ≥0.53 on factor 1 (“Team quality & communication”) for items 22, 23, 25, 26, 28, 29, and 32; ≥0.40 on factor 2 (“Family impact”) for items 33, 34, 35, and 27; and ≥0.71 on factor 3 (“Access to care”) for items 17 and 18. All factor loadings can be found in Table 3.

**Table 3:** Exploratory factor analysis of psychometrically tested, Likert-scaled PICS items.

No. of item in original PICS	Translated item wording of the PICS-D	Factor 1	Factor 2	Factor 3
22	How often have the care network members explained things to you in a way that you could understand?	*0*.*63*	−0.11	−0.03
23	How often did you feel that treatment recommendations were passed between the members of the care network?	*0*.*53*	0.22	0.01
25	How often did you feel that the members of the care network listened to what you had to say about your child's health?	*0*.*88*	−0.06	0.10
26	How often has someone explained to you who was responsible for different parts of your child's care?	*0*.*61*	0.28	0.00
28	How often did you feel that the care network members were fulfilling their roles in your child's care?	*0*.*78*	−0.05	−0.08
29	How often have you felt that care network members thought about the “big picture” when caring for your child, meaning dealing with all of your child's needs?	*0*.*61*	0.20	−0.12
32	How often have the care network members treated you as a full partner?	*0*.*68*	0.02	−0.11
33	How often have the care network members talked with you about how care decisions for your child will affect your whole family?	0.15	*0*.*70*	−0.12
34	How often have members of the care network talked with you about burdens you may face as a result of your child's illness?	0.01	*0*.*92*	−0.02
35	How often have the care network members addressed situations that may make it difficult for you to care for your child's health (e.g., work, own illness, etc.)?	−0.04	*0*.*91*	0.07
37	How often have the members of the care network offered you the opportunity to connect with other affected families?	0.05	*0*.*40*	−0.02
17	How often did you have difficulties getting medical or social services (e.g., therapies, integration assistance, etc.), because there were waiting lists or other problems getting appointments?	0.07	−0.04	*0*.*77*
18	How often did you have difficulties getting medical or social services (e.g., therapies, integration assistance, etc.), because you did not know who to contact?	−0.11	0.06	*0*.*71*

We tested the model fit of the congeneric model for factors 1 and 2. The congeneric model is the least restrictive model of classical test theory, in which all items belong to one latent dimension. The fit indices suggested a good fit between the model and the data for factor 1 (CFI = 0.96, TLI = 0.94, RMSEA = 0.10) and a reasonable fit for factor 2 (CFI = 0.99, TLI = 0.96, RMSEA = 0.12). Since factor 3 consists of only two items, we used a parallel model to test unidimensionality because less restrictive models require more items. The parallel model combines the characteristics of the congeneric model with the assumption that correlations and measurement errors should be the same above all items. The fit indices suggested a good fit between the model and data for factor 3 (CFI = 1.00, TLI = 1.00, RMSEA < 0.01). Examining the entire three-factorial model, the fit indices suggested a good fit between the model and the data (CFI = 0.95, TLI = 0.94, RMSEA = 0.07).

Looking at the content of the items loading on factor 1, we observed that the focus was primarily on the interaction between families and health care providers. Simultaneously, factor 1 included items on the quality of communication in the care network. Therefore, we named factor 1 “Team quality & communication”. Items that loaded on factor 2 mainly addressed the impact of care (decisions) on families' daily lives. For this reason, we designated factor 2 “Family impact”. Factor 3 consisted of two items that concerned the access to care, so we named this factor “Access to care”.

### Reliability

Cronbach's *α* was 0.89 and 0.84 in factors 1 and 2, respectively. Due to the absence of essential tau-equivalence in the model test, Cronbach's *α* is not interpretable as a reliability measure. We thus also report McDonald's *ω_t_*. McDonald's *ω_t_* was 0.93 and 0.88 in factors 1 and 2, respectively. Since factor 3 contains only two items, we could not calculate Cronbach's *α* or McDonald's *ω_t_*. As the inter-item correlation with *r* = 0.55 can be used as a reliability value, this factor shows insufficient reliability.

## Discussion

In this study, we developed and psychometrically tested a German version of the Pediatric Integrated Care Survey (PICS), the so-called PICS-D. We can provide evidence that the PICS-D is a reliable and valid instrument by which to assess the experiences of IC as reported by the caregivers of chronically ill children. It takes a multidimensional approach and explores several crucial aspects of IC, categorized into three factors. Factor 1 “Team quality & communication” reflects the perceived quality of care and the cooperation within the care network (between all persons involved in caring for the chronically ill child in the last 12 months at the time of reporting), e.g., exchange of treatment recommendations, fulfillment of tasks involved in the care (2). Moreover, factor 1 analyzes the experiences concerning the communication between healthcare professionals and caregivers and the degree of the caregiver's involvement in decisions concerning the child's health, e.g., the feeling of being treated as a full partner (6, 22). Factor 2 “Family impact” evaluates the influence of care decisions and interventions on the entire family, e.g., burdens arising from the child's illness, opportunities for networking with other affected families (34, 35). Factor 3 “Access to care” investigated key elements of access to care, e.g., difficulties coordinating appointments (4, 36). Due to the insufficient reliability of the third PICS-D factor, we recommend using only factors 1 and 2 for scoring the PICS-D. To address this issue, it is necessary to develop a new associated item pool to re-examine this factor *via* factor analysis. More than four items would be desirable to perform in-depth statistical analyses. To obtain a rudimentary comparison with the psychometric properties of the original PICS, we used Cronbach's *α* for the two respective largest factors (21). The first PICS-D factor's internal consistency (“Team quality & communication”; Cronbach's *α *= 0.89) is slightly higher compared to the first original PICS factor (“Communication between health care provider and parent”; Cronbach's *α *= 0.80). In addition, the second PICS-D factor's internal consistency (“Family impact”; Cronbach's *α *= 0.84) is higher compared to the second original PICS factor (“Family impact”, Cronbach's *α *= 0.72).

The project team reviewed the items excluded from the analyses at both methodological and content levels. They seem to be well captured by the other items' content in the factors. A possible explanation for misunderstanding an item's wording could be the translation: In German, items tend to be longer and less commonly used, possibly triggering different latent constructs. However, the recovered factor structure in PICS-D appears to be topically similar to the original PICS’ factor structure (except for the excluded items) (11, 21). Factors 2 and 3 consist of identical items such as the “Access to care”- and “Family impact”-factors in the original PICS. Factor 1 represents a composite of the original factors “Communication between health care provider and parent” and “Team functioning/performance/quality/connectivity”.

Several strengths must be underlined. As far as we know, the PICS-D is the first and currently only German instrument by which to assess the perceived IC quality reported by caregivers of chronically ill children. This study examined its psychometric properties using a relevant nationwide sample. Our project provides compelling support for the critical importance of the role of patients and families in the design of health care delivery models, and in the evaluation of the performance of those models (37). The PICS-D is an initial step toward evaluating possible IC interventions to improve care for chronically ill children and increase caregivers' involvement in the care process. We used rigorous pre- and post-data collection procedures to enable the PICS' transferability to the German healthcare context (extensive translation process; two phases of cognitive interviews with intermittent and final revisions of the PICS-D; multiple virtual meetings with original authors to ensure original meanings of items; combining methodological and qualitative considerations in excluding items, etc.).

Our study has some limitations. Our sample size is at the lower limit of feasibility for psychometric testing. However, through intensive data management, we were able to ensure that the number of missing values was kept to a minimum to guarantee the feasibility of all our statistical analyses. Our assessment of the questionnaire's additional psychometrically important properties (e.g., other facets of construct validity, criterion validity, test-retest reliability, etc.) requires further methodological efforts. It is therefore essential to replicate the questionnaire's structure working with other samples, especially with regard to the factor structure, since factors with only two items are statistically problematic and should only interpreted with care (38, 39). The original PICS was developed in the US healthcare system context by relying on interviews and focus groups with patients and caregivers. The scanned literature contained various definitions of care integration (2, 4, 6, 8, 11–13, 20, 21, 37). Although these are similar in essence, we could not rule out that different latent IC facets are triggered when answering the PICS-D. To design a valid measurement instrument, it is essential to develop a profound theoretical basis. Further psychometric testing in other countries is needed to refine a theoretical IC framework in pediatric settings by considering different experiences in international health contexts. Our study may support the further development of just such a framework.

## Conclusion

The PICS-D is the first German-language questionnaire to assess the integration of pediatric care. Thanks to this study's positive findings, we can recommend the use of the PICS-D. Ziniel and co-authors describe a broad range of applications for the original PICS (21). Accordingly, the PICS-D can also be used to identify gaps in care delivery, guide quality improvement efforts, or measure the impact of a care plan or care model. Used under consideration of its framework, the results generated from our study reveal benefits for researchers, decision-makers, and field practitioners alike.

Principal axis factoring after direct oblimin rotation. Item information was summarized in 3 factors: Team quality & communication (factor 1), Family impact (factor 2), and Access to care (factor 3). Values are standardized factor loading: a higher value indicates a strong correlation with the corresponding factor. Values greater or equal 0.40 are in italics.

## Data Availability

The original contributions presented in the study are included in the article/Supplementary Material, further inquiries can be directed to the corresponding author/s.

## References

[1] Gerlach FM Schaeffer D Greiner W Thürmann PA Wille E Haubitz M Wettbewerb an der schnittstelle zwischen ambulanter und stationärer gesundheitsversorgung. Bericht des sachverständigenrates zur begutachtung der entwicklung im gesundheitswesen. Bonn: NOMOS; 2012 (2012).

[2] Sterly C Hassler M. Integrierte versorgung. In: Thielscher CH, editors. Medizinökonomie. Wiesbaden: Gabler Verlag | Springer Fachmedien (2012). p. S.483–504.

[3] Mühlbacher A. Integrierte versorgung. Management und organisation. Hans Huber: Bern u.a. (2002).

[4] Wolfe I Lemer C Cass H. Integrated care: a solution for improving children's Health? Arch Dis Child. (2016) 101(11):992–7. 10.1136/archdischild-2013-304442 27052949

[5] World Health Organisation. Integrated care models: An overview. Geneva, Switzerland: World Health Organisation (2016). Verfügbar unter: www.euro.who.int/__data/assets/pdf_file/0005/322475/Integrated-caremodels- overview.pdf

[6] Singer SJ Burgers J Friedberg M Rosenthal MB Leape L Schneider E. Defining and measuring integrated patient care: promoting the next frontier in health care delivery. Med Care Res Rev. (2011) 68(1):112–27. 10.1177/1077558710371485 20555018

[7] Goodwin N. Understanding integrated care. Int J Integr Care. (2016) 16(4):1–4. 10.5334/ijic.2530. Verfügbar unter: https://www.ncbi.nlm.nih.gov/pmc/articles/PMC5354214/PMC5354214 28316546

[8] Wolfe I Satherley RM Scotney E Newham J Lingam R. Integrated care models and child health: a meta-analysis. Pediatrics. (2020) 145(1):1–12. 10.1542/peds.2018-3747. Verfügbar unter: https://pediatrics.aappublications.org/content/145/1/e20183747 31888959

[9] Armitage GD Suter E Oelke ND Adair CE. Health systems integration: state of the evidence. Int J Integr Care. (2009) 9(2):1–11. 10.5334/ijic.316. Verfügbar unter: https://www.ncbi.nlm.nih.gov/pmc/articles/PMC2707589/PMC2707589 19590762

[10] Schor EL. American Academy of pediatrics task force on the family. Family pediatrics: report of the task force on the family. Pediatrics. (2003) 111(6 Pt 2):1541–71. 12777595

[11] Antonelli R. Pediatric integrated care survey 1.0 user manual. Boston: Boston Children's Hospital (2015).

[12] Antonelli R Mcallister J Popp J. Making care coordination a critical component of the pediatric health system: a multidisciplinary framework (2009). Verfügbar unter: https://www.commonwealthfund.org/publications/fund-reports/2009/may/making-care-coordination-critical-component-pediatric-health

[13] Turchi R Antonelli R Norwood K Adams R Brei T. Burke R. Patient-and family-centered care coordination: a framework for integrating care for children and youth across multiple systems. Pediatrics. (2014) 133:E1451–60. 24777209 10.1542/peds.2014-0318

[14] Eikötter T Greiner W. Instrumente zur messung der versorgungsqualität in der integrierten versorgung. Gesundheitsökonomie Qualitätsmanagement. (2008) 13(1):25–31. 10.1055/s-2007-963333

[15] Wolfe I. Health services for children with long-term conditions and non-communicable diseases. In: Wolfe I McKee M Herausgeber, editors. European Child health services and systems: lessons without borders. Maidenhead: Open University Press (2013). p. S.63–93.

[16] Weatherly JN Lägel R Herausgeber. Neue versorgungsansätze in der psychiatrie, neurologie und psychosomatik. Berlin: Medizinisch Wissenschaftliche Verlagsgesellschaft (2009).

[17] Asarnow JR Rozenman M Wiblin J Zeltzer L. Integrated medical-behavioral care compared with usual primary care for child and adolescent behavioral health: a meta-analysis. JAMA Pediatr. (2015) 169(10):929–37. 10.1001/jamapediatrics.2015.1141 26259143

[18] Latour JM Duivenvoorden HJ Tibboel D Hazelzet JA, EMPATHIC Study Group. The shortened EMpowerment of PArents in THe intensive care 30 questionnaire adequately measured parent satisfaction in pediatric intensive care units. J Clin Epidemiol. (2013) 66(9):1045–50. 10.1016/j.jclinepi.2013.02.010 23790723

[19] Girch A Rippe RCA Latour JM Jönebratt Stocker M Blendermann M Hoffmann K. The German EMPATHIC-30 questionnaire showed reliability and convergent validity for use in an intermediary/general pediatric cardiology unit: a psychometric evaluation. Front Cardiovasc Med. (2022) 9:901260. 10.3389/fcvm.2022.901260 35811695 PMC9262329

[20] Cohen E Coller RJ. Evaluating integrated care for children: a clarion call or a call for clarity? Pediatrics. (2020) 145(1):1–2. 10.1542/peds.2019-3282. Verfügbar unter: https://pediatrics.aappublications.org/content/145/1/e20193282 31888958

[21] Ziniel SI Rosenberg HN Bach AM Singer SJ Antonelli RC. Validation of a parent-reported experience measure of integrated care. Pediatrics. (2016) 138(6):1–11. 10.1542/peds.2016-0676 27940672

[22] Noest S Ludt S Klingenberg A Glassen K Heiss F. Ose D. Involving patients in detecting quality gaps in a fragmented healthcare system: development of a questionnaire for Patients’ experiences across health care sectors (PEACS). Int J Qual Health Care. (2014) 26(3):240–9. 10.1093/intqhc/mzu044 24758750 PMC4041096

[23] Hildebrandt H Pimperl A Schulte T Hermann C Riedel H. Schubert I. Triple aim—evaluation in der integrierten versorgung gesundes kinzigtal—gesundheitszustand, versorgungserleben und wirtschaftlichkeit. Bundesgesundheitsbl. (2015) 58(4):383–92. 10.1007/s00103-015-2120-y 25652116

[24] Klingenberg A Bahrs O Szecsenyi J. How do patients evaluate general practice? German results from the European project on patient evaluation of general practice care (EUROPEP). Zeitschrift für ärztliche Fortbildung und Qualitätssicherung. (1999) 93:437–45. 10519193

[25] Braun S Kreimeier S Greiner W. Messung der patientenzufriedenheit in der integrierten versorgung—eine pilotstudie mit dem modifizierten ZAP-fragebogen. Zeitschrift für evidenz. Fortbildung und Qualität im Gesundheitswesen. (2010) 104(2):106–12. 10.1016/j.zefq.2009.12.001 20441017

[26] Bethge M Bartel S Streibelt M Lassahn C Thren K. Verbesserte behandlungsqualität durch integrierte versorgung bei knie- und hüftgelenkersatz: ergebnisse einer kontrollierten studie. Rehabilitation. (2011) 50(2):86–93. 10.1055/s-0030-1265144 21503861

[27] Willems J Farin-Glattacker E Langer T. Evaluation of a case management to support families with children diagnosed with spinal muscular atrophy—protocol of a controlled mixed-methods study. Front Pediatr. (2021) 9:614512. 10.3389/fped.2021.614512 34414138 PMC8369478

[28] Eremenco SL Cella D Arnold BJ. A comprehensive method for the translation and cross-cultural validation of health status questionnaires. Eval Health Prof. Juni. (2005) 28(2):212–32. 10.1177/0163278705275342 15851774

[29] Willis GB. Cognitive interviewing: a tool for improving questionnaire design. California, USA: SAGE Publications (2004). 375 S.

[30] R Core Team. R: a language and environment for statistical computing. Vienna, Austria: R Foundation for Statistical Computing (2020). Verfügbar unter: https://www.R-project.org/

[31] Velicer WF. Determining the number of components from the matrix of partial correlations. Psychometrika. (1976) 41:321–7. 10.1007/BF02293557

[32] Hooper D Coughlan J Mullen M. Structural equation modeling: guidelines for determining model fit. Electron J Bus Res Methods. (2007) 30:6.

[33] Hu Lt Bentler PM. Cutoff criteria for fit indexes in covariance structure analysis: conventional criteria versus new alternatives. Struct Equ Modeling Multidiscip J. (1999) 6(1):1–55. 10.1080/10705519909540118

[34] Hjorth E Kreicbergs U Sejersen T Jeppesen J Werlauff U Rahbek J. Bereaved parents more satisfied with the care given to their child with severe spinal muscular atrophy than nonbereaved. J Child Neurol. (2019) 34(2):104–12. 10.1177/0883073818811544 30518279

[35] Mastellos N Gunn L Harris M Majeed A Car J Pappas Y. Assessing patients’ experience of integrated care: a survey of patient views in the north west London integrated care pilot. Int J Integr Care. (2014) 14:1–9. 10.5334/ijic.1453 PMC4059210 24987321

[36] Altman L Zurynski Y Breen C Hoffmann T Woolfenden S. A qualitative study of health care providers’ perceptions and experiences of working together to care for children with medical complexity (CMC). BMC Health Serv Res. (2018) 18(1):70. 10.1186/s12913-018-2857-8 29386026 PMC5793356

[37] Bautista MAC Nurjono M Lim YW Dessers E Vrijhoef HJ. Instruments measuring integrated care: a systematic review of measurement properties. Milbank Q. (2016) 94(4):862–917. 10.1111/1468-0009.12233 27995711 PMC5192798

[38] Marsh HW Hau KT Balla JR Grayson D. Is more ever too much? The number of indicators per factor in confirmatory factor analysis. Multivariate Behav Res. (1998) 33(2):181–220. 10.1207/s15327906mbr3302_1 26771883

[39] Yong A Pearce S. A Beginner's guide to factor analysis: focusing on exploratory factor analysis. Tutor Quant Methods Psychol. (2013) 9:79–94. 10.20982/tqmp.09.2.p079

